# Inflammatory mechanisms in diabetic nephropathy: emerging insights and targeted therapeutics

**DOI:** 10.3389/fmed.2025.1722159

**Published:** 2026-01-16

**Authors:** Shengnan Lin, Zheng Shu, Mi Zhou, Zhen Jia, Tianshu Wei, Xiaojun Zhou

**Affiliations:** 1 Department of Endocrinology and Metabology, The First Affiliated Hospital of Shandong First Medical University & Shandong Provincial Qianfoshan Hospital, Shandong Institute of Nephrology, Jinan, Shandong, China; 2 Shandong First Medical University & Shandong Academy of Medical Sciences, Jinan, Shandong, China; 3 Department of Vascular Surgery, Beijing Jishuitan Hospital, Capital Medical University, Beijing, China; 4 Department of Clinical Laboratory Medicine, The First Affiliated Hospital of Shandong First Medical University & Shandong Provincial Qianfoshan Hospital, Jinan, Shandong, China; 5 State Key Laboratory of Discovery and Utilization of Functional Components in Traditional Chinese Medicine, School of Pharmaceutical Sciences, Cheeloo College of Medicine, Shandong University, Jinan, Shandong, China; 6 Drug Discovery Biology, Monash Institute of Pharmaceutical Sciences, Monash University, Parkville, VIC, Australia

**Keywords:** diabetic kidney disease, GLP-1 receptor agonists, inflammation, multi-omics, NLRP3 inflammasome, non-steroidal mineralocorticoid receptor antagonists, pathogenesis, SGLT2 inhibitors

## Abstract

**Background:**

Diabetic kidney disease (DKD) remains a leading cause of end-stage renal disease (ESRD) worldwide. Understanding of DKD pathogenesis has undergone a pivotal shift, moving beyond traditional metabolic and hemodynamic paradigms to underscore the critical role of chronic inflammation.

**Objective:**

This review aims to systematically delineate recent advances in the inflammatory mechanisms of DKD and to discuss their translational implications. It will focus on emerging diagnostic biomarkers and novel inflammation-targeted therapeutic strategies.

**Main content:**

This review portrays the complex interplay of emerging inflammatory mechanisms in DKD, encompassing inflammatory pathway activation, cellular senescence, impaired podocyte autophagy, the gut microbiota-kidney axis, and regulation by non-coding RNAs (ncRNAs). Meanwhile, a novel diagnostic paradigm powered by omics technologies and artificial intelligence (AI) is described, highlighting the associated biomarkers. Lastly, the therapeutic landscape, focusing on agents with proven renal benefits, including sodium-glucose cotransporter 2 (SGLT2) inhibitors, non-steroidal mineralocorticoid receptor antagonists (ns-MRAs), and glucagon-like peptide-1 receptor agonists (GLP-1RAs) is reviewed, with evaluating the promise of natural products as multi-target interventions.

**Conclusion:**

Inflammation in DKD is driven by an intricate network of local and systemic factors. A multifaceted approach which prioritizes the integration of multi-omics data for inflammatory subtyping, deciphering inter-organ communication, and developing combined therapies that leverage conventional drugs, targeted agents, and natural compounds should be adopted to advance the management of DKD.

## 1 Introduction

Diabetic kidney disease (DKD) is a leading cause of chronic kidney disease (CKD) and end-stage renal disease (ESRD) worldwide, affecting approximately 20%−40% of individuals with diabetes (1). As the global prevalence of diabetes continues to rise, the burden of DKD increases substantially. It poses a major public health challenge that severely compromises patients' quality of life and survival (2). Traditionally, the pathogenesis of DKD has been attributed to two core mechanisms, metabolic dysregulation and hemodynamic abnormalities (3, 4). Hyperglycemia induces renal cell injury through multiple metabolic pathways, including activation of the polyol pathway, formation of advanced glycation end products (AGEs), activation of protein kinase C (PKC), and upregulation of the hexosamine pathway. Concurrently, hemodynamic changes—particularly dilation of afferent arterioles and constriction of efferent arterioles—create a state of high perfusion, high pressure, and high filtration (the “triple high” state) within the glomerulus. “Triple high” state subsequently accelerates glomerulosclerosis and functional deterioration (5, 6). These two mechanisms intertwine to form the classic pathophysiological foundation of DKD.

However, the clinical diagnosis and management of DKD remain difficult. Although renin–angiotensin–aldosterone system (RAAS) inhibitors are widely used as the cornerstone therapy, a significant “ceiling effect” is observed, with many patients continuing to experience disease progression (7). Moreover, DKD exhibits considerable heterogeneity, and its pathogenesis extends beyond the traditional metabolic and hemodynamic paradigms. Current diagnostic approaches, which rely on urinary protein and estimated glomerular filtration rate (eGFR), often detect abnormalities only after irreversible structural kidney damage has occurred, thereby missing the optimal window for intervention (8). These clinical challenges emphasize the urgent need to explore pathogenic mechanisms beyond the traditional metabolic and hemodynamic paradigms. In this context, chronic low-grade inflammation has emerged as a crucial mechanism spanning the entire course of DKD.

Accumulating evidence indicates that chronic inflammation, triggered jointly by hyperglycemia, AGEs, and hemodynamic stress, interacts with fibrosis, cellular senescence, and other emerging pathways to form a complex pathophysiological network in DKD. Exploring these novel mechanisms and develop corresponding early diagnostic tools and targeted therapies is essential for overcoming current bottlenecks in DKD management. Notably, the pathogenesis of DKD should not be viewed as an isolated progress in the kidney. Diabetes is fundamentally a systemic metabolic disorder characterized by a chronic low-grade inflammatory state, with complications exhibiting typical panvascular features. Therefore, DKD can be regarded as the renal manifestation of diabetic panvascular disease, sharing common pathological features—such as endothelial dysfunction, oxidative stress, and chronic inflammation—with diabetic cardiovascular disease and retinopathy (9). This perspective highlights the intrinsic connections among organ-specific complications and provides a theoretical foundation for therapies with dual cardiorenal benefits, such as sodium–glucose cotransporter 2 (SGLT2) inhibitors. Furthermore, renal inflammatory injury arises not only from the activation of intrinsic renal cells but also remotely from extrarenal chronic inflammatory conditions, such as periodontitis, via “organ–organ crosstalk” (10).

**Figure 1:**
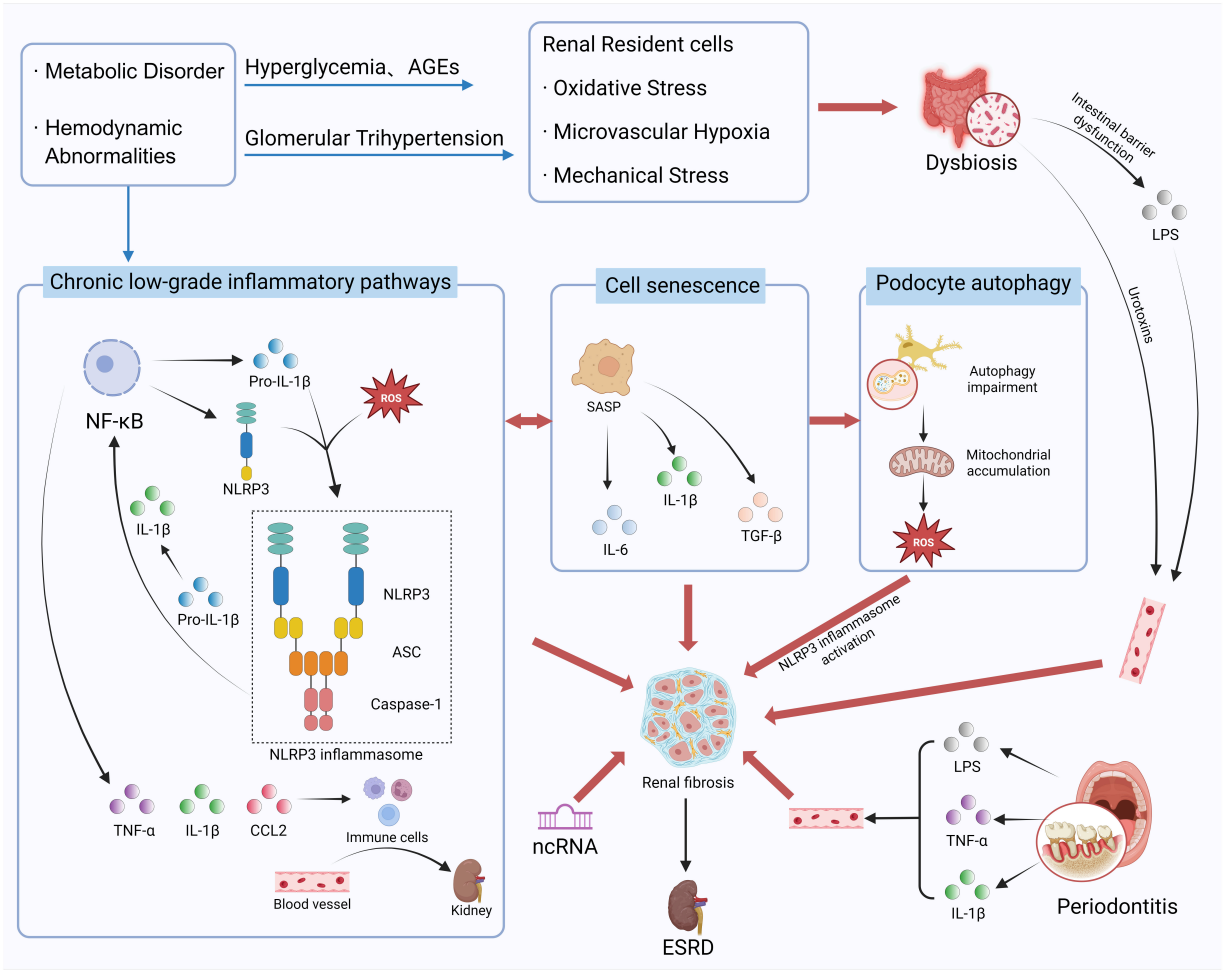
The complex pathogenesis network of diabetic kidney disease (DKD). Created with BioRender.com.

Inflammation in DKD constitutes a complex network driven by both local and systemic factors (Figure 1). This review will delineate key inflammatory mechanisms—including the activation of inflammatory pathways, cellular senescence, impaired podocyte autophagy, gut microbiota-kidney axis, and non-coding RNAs (ncRNAs) regulation—within the broader context of systemic inflammation and panvascular disease. It will also systematically characterize recent advance in diagnostic technologies based on multi-omics and artificial intelligence (AI). Last but not the least, emerging therapeutic strategies such as SGLT2 inhibitors, non-steroidal mineralocorticoid receptor antagonists (ns-MRAs) (e.g., Finerenone), glucagon-like peptide-1 receptor agonists (GLP-1RAs), and natural products with multi-target potential will be summarized and discussed. Overall, this review aims to bridge bench-side discoveries with bedside applications, providing a translational perspective for the development of precision medicine in DKD management.

## 2 Emerging pathogenesis

### 2.1 Inflammatory pathway activation

Persistent hyperglycemia, metabolic dysregulation (e.g., AGEs), and hemodynamic alterations collectively stimulate intrinsic renal cells (e.g., podocytes, mesangial cells) and induce microvascular hypoxia (11). These stress signals, through pattern recognition receptors (e.g., TLRs), activate classic inflammatory pathways such as NF-κB and JAK/STAT (12, 13). Subsequently, local renal production of chemokines (e.g., CCL2) and pro-inflammatory factors (e.g., IL-1β, TNF-α) (14) recruit immune cells like monocytes/macrophages and directly damage the glomerular filtration barrier, inducing tubular epithelial cell transdifferentiation (3). Crucially, pro-inflammatory factors also function as potent pro-fibrotic mediators. They directly up-regulate the expression of transforming growth factor-β1 (TGF-β1) in renal resident cells and activate its downstream Smad signaling pathway, tightly coupling inflammation with fibrosis (15). The NOD-like receptor family pyrin domain containing 3 (NLRP3) inflammasome, comprising NLRP3, caspase-1, and apoptosis-associated speck-like protein containing a caspase recruitment domain (ASC), has been identified as a pivotal hub linking metabolic risk factors to the formation and release of IL-1β (16). Furthermore, abnormal activation of the adaptive immunity, particularly involving Th17 cells, exacerbates tissue injury (17). Chronic, low-grade inflammation and associated renal fibrosis represent a key mechanism in DKD progression.

Importantly, inflammatory pathways outlined above do not operate in isolation, they form a tightly interwoven and mutually amplifying regulatory network. Among these, the crosstalk between NF-κB and the NLRP3 inflammasome is particularly critical. NF-κB activation provides the “priming” signal for NLRP3 inflammasome activation by upregulating the transcriptional expression of NLRP3 and pro-IL-1β, preparing the ground for inflammasome assembly. Subsequently, upon a second signal [e.g., ATP, reactive oxygen species (ROS), or crystalline substances], the NLRP3 inflammasome becomes fully activated, catalyzing the cleavage of pro-IL-1β into mature IL-1β and facilitating its massive release. IL-1β, in turn, acts as a potent pro-inflammatory cytokine that can further trigger NF-κB signaling, forming a positive feedback loop that dramatically amplifies the inflammatory response (18–20). Notably, the precise hierarchical relationships and relative contributions of this crosstalk within different renal resident cells and infiltrating immune cells remain incompletely understood. Therefore, deciphering this regulatory network at a cell-specific level represents a key challenge and a priority for future research in this field.

Beyond intrarenal inflammation, systemic chronic inflammation also significantly contributes to DKD progression. A prime example is the inflammatory crosstalk along the oral-systemic axis. Periodontitis, a common chronic inflammatory oral disease, harbors a dysbiotic microbiome that serves as a persistent source of pro-inflammatory mediators. This reservoir releases factors such as lipopolysaccharides (LPS), IL-1β, and TNF-α into the circulation, exacerbating the systemic inflammatory burden in diabetic patients. Circulating inflammatory factors subsequently aggravate renal endothelial dysfunction and inflammatory responses, establishing an “oral-renal axis” that accelerates DKD progression (21–23). Hence, renal inflammation in DKD is an integrated process driven by both local and systemic factors.

### 2.2 Cellular senescence

Senescent cells refer to intrinsic renal cells that enter an irreversible state of growth arrest following prolonged exposure to stressors such as high glucose levels (24). Unlike traditional apoptosis, senescent cells do not die but instead transform into a senescence-associated secretory phenotype (SASP), continuously releasing large amounts of inflammatory mediators, chemokines, and pro-fibrotic factors. Hence, they create a chronic low-grade inflammatory and fibrotic network within the local microenvironment (25)^.^ This state not only directly impairs renal cell function (e.g., podocyte detachment), but also “contaminates” the surrounding healthy cells through paracrine effects, accelerating glomerulosclerosis, and renal interstitial fibrosis (26). Thus, cellular senescence is regarded as a critical bridge linking metabolic stress to the clinical-pathological alterations in DKD. “Anti-senescence therapies” facilitating senescent cell clearance have emerged as a highly promising new strategy for DKD.

### 2.3 Impaired podocyte autophagy

As highly differentiated terminal cells, podocytes are central to maintaining the glomerular filtration barrier. Injury or loss of podocytes represents a key event in glomerular filtration barrier disruption. In recent years, autophagy has gained attention as a critical mechanism for preserving podocyte homeostasis. Autophagy enables cells to degrade damaged components under stress, thereby maintaining energy balance and supporting intracellular quality control (27). Under diabetic conditions, however, persistent hyperglycemia, metabolic dysregulation, and oxidative stress suppress autophagic flux in podocytes. Consequently, dysfunctional organelles, such as damaged proteins and mitochondria, accumulate due to ineffective clearance (28). In the meantime, impaired autophagy promotes podocyte apoptosis, epithelial-mesenchymal transdifferentiation, and detachment, ultimately compromising the structural and functional integrity of the filtration barrier (29). Furthermore, autophagy-deficient podocytes accumulate damaged mitochondria, resulting in excessive ROS production that in turn activates inflammatory pathways, including the NLRP3 inflammasome described above (30–32). This mechanism closely links metabolic stress to local renal inflammation. Therefore, restoring podocyte autophagy has emerged as a promising therapeutic strategy for DKD. Interventions aimed at enhancing autophagic activity—whether by pharmacological or genetic means—may help to clear cytotoxic aggregates and protect podocytes from hyperglycemia-induced injury.

### 2.4 Gut microbiota-kidney axis

The gut microbiota-kidney axis has emerged as a concept of considerable interest in understanding the pathogenesis of DKD, suggesting a bidirectional communication network between the intestinal microbial ecosystem and renal physiology. In diabetic conditions, persistent hyperglycemia and dietary modifications frequently drive gut microbiota dysbiosis which is characterized by a diminished abundance of beneficial bacteria and an expansion of opportunistic pathogens (33). Several interconnected mechanisms are proposed for renal injury contributed by gut microbiota dysbiosis.

Firstly, dysbiotic microbiota enhance the production of harmful metabolites, including indole-3-sulfate and p-cresol sulfate. Upon entering the systemic circulation, these uremic toxins accumulate in the kidneys, where they promote oxidative stress, inflammation, and fibrosis (34). Secondly, dysbiosis impairs intestinal barrier function, facilitating the translocation of endotoxins such as LPS into the bloodstream. Consequently, persistent, low-grade systemic inflammation that accelerates DKD progression is triggered (35). Moreover, declining renal function further impedes the excretion of harmful metabolites and endotoxins, intensifying dysbiosis and establishing a self-sustaining vicious cycle. Targeted modulation of the gut microbiota—through prebiotics, probiotics, or dietary intervention—has gained attention as a promising therapeutic strategy to slow DKD progression by acting upon this mechanism. Several natural products have shown potential in modulating this axis. For example, studies indicate that medicinal herbs such as rhubarb and astragalus can reshape gut microbial composition. Subsequent reduction of uremic toxins mitigates renal inflammation and oxidative injury (36, 37). Evidence for promising treatment suggests the gut-kidney axis is a pivotal and actionable target in DKD.

### 2.5 Regulation by ncRNAs

The regulatory roles of ncRNAs constitute an emerging and rapidly advancing frontier in DKD pathogenesis research. Among them, microRNAs (miRNAs), long non-coding RNAs (lncRNAs), and circular RNAs (circRNAs) collectively establish a complex regulatory network. miRNAs such as miR-21, miR-29a, and miR-802 contribute to the regulation of inflammation, fibrosis, and apoptosis in renal cells through direct targeting of key mRNAs (38–40). For example, elevated miR-802 expression is associated with renal inflammation and fibrosis. Hence, miR-802 has the potential to be used as both diagnostic biomarker and therapeutic target. LncRNAs and circRNAs frequently operate as competitive endogenous RNAs that function as “molecular sponges,” sequestering specific miRNAs and thereby alleviating miRNA-mediated repression of target genes to modulate disease progression (41). In DKD, several circRNAs—including circRNA LDL receptor related protein 6 and circ_0000064—have been implicated in mediating podocyte injury, mesangial cell hypertrophy, and tubulointerstitial fibrosis via regulation of miRNA and protein expression (42). To elaborate specific roles of lncRNAs, up-regulation of lncRNA ISG20 exacerbates renal fibrosis (43). In the meantime, lncRNA NEAT1 promotes tubular epithelial cell injury through regulation of mitophagy (43). Furthermore, downregulation of lncRNA MALAT1 confers protection to podocytes under hyperglycemic conditions (43). Owing to their tissue-specific expression and detectability, ncRNAs not only provide novel insights into pathogenesis of DKD but also exhibit considerable promise as early biomarkers and targets for RNA-based therapeutics.

## 3 Cutting-edge diagnostic technologies and biomarkers

In recent years, the diagnosis and treatment of DKD have entered a new era of precision medicine. On one hand, leveraging high-throughput technologies such as proteomics and metabolomics, researchers have successfully identified multiple novel biomarkers (such as specific urinary protein fragments and circulating metabolites) that hold promise for earlier, more specific risk prediction and disease staging (44, 45). Simultaneously, AI technologies are being deeply integrated into renal imaging analysis, enabling intelligent recognition of subtle pathological features within medical images and significantly enhancing diagnostic efficiency and objectivity (46, 47). The convergence of these cutting-edge technologies collectively advances the early detection and personalized intervention of DKD.

### 3.1 Omics-based novel biomarkers

The rapid advancement of high-throughput omics technologies has revolutionized early diagnosis and precision intervention in DKD. Unlike traditional approaches that rely on relatively delayed indicators, proteomics and metabolomics enable unbiased, systematic screening of thousands of molecular alterations in patients' biofluids (e.g., urine and blood), which reveals disease-specific signatures at earlier pathological stages (48, 49). For instance, proteomic studies have identified specific collagen degradation fragments (e.g., type III collagen C-terminal peptides) and extracellular matrix-related proteins in urine, changes in which often precede the clinical detection of microalbuminuria and signal the initiation of renal fibrosis (50). Metabolomics, on the other hand, elucidates complex disturbances in systemic energy metabolism networks. It identifies gut microbiota-derived metabolites such as indoxyl sulfate and p-cresyl sulfate. It also detects disruptions in carnitine metabolism and TCA cycle intermediates linked to mitochondrial dysfunction. These molecules not only serve as indicators of renal injury but may also actively contribute to disease pathogenesis (51, 52).

The value of these novel omics-derived biomarkers extends beyond ultra-early risk prediction. They also help define distinct molecular subtypes of DKD, providing a robust scientific basis for personalized prognosis assessment, targeted drug development, and dynamic monitoring of therapeutic response. Together, these capabilities are steering the field toward the era of precision medicine. Importantly, the molecular subtypes delineated by these biomarkers frequently correlate with the activation of specific inflammatory pathways. For example, certain biomarker profiles may indicate an inflammatory subtype dominated by the NLRP3-IL-1β axis, whereas others may reflect inflammatory patterns driven by TNF-α or CCL2 (53). Omics-guided “inflammatory subtyping” strategy establishes a theoretical foundation for achieving precision anti-inflammatory therapy tailored to specific inflammatory cascades. A summary of representative novel biomarkers discovered via omics technologies is provided in Table 1.

**Table 1:** Representative novel biomarkers for DKD discovered by omics technologies.

**Biomarker category**	**Specific examples**	**Source**	**(Patho)physiological significance/associated pathways**	**Clinical potential**	**References**
Protein	C-terminal propeptide of type III collagen and other extracellular matrix (ECM) fragments	Urine	Reflects early activation of renal fibrogenesis and ECM turnover; fibrosis is closely associated with TGF-β signaling and chronic inflammation.	Serves as an early biomarker of fibrosis, potentially detectable before the onset of microalbuminuria.	(50)
Metabolite	Indoxyl sulfate, p-cresyl sulfate	Serum/Urine	Gut microbiota-derived uremic toxins; induce oxidative stress and activate inflammatory pathways such as the NLRP3 inflammasome and NF-κB.	Connects the “gut-kidney axis”; potential utility for risk stratification and monitoring response to interventions.	(51)
Metabolite	TCA cycle intermediates, Acylcarnitines	Serum/Urine	Indicates mitochondrial dysfunction and impaired fatty acid oxidation; mitochondrial damage is a key source of reactive oxygen species (ROS) and can trigger inflammation (e.g., via NLRP3).	Aids in identifying metabolic subtypes of DKD and may improve prognostic evaluation.	(52)

### 3.2 Applications of bioinformatics and AI in diagnosis

The synergistic application of bioinformatics and AI is advancing diagnosis of DKD through several distinct technical pathways. In bioinformatics, it is demonstrated through the in-depth analysis of multi-omics data. Integration of genomic, metabolomic, and proteomic datasets enables the construction of molecular interaction networks. For example, KEGG and GO enrichment analyses help identify fibrosis-related pathways and energy metabolism pathways that are activated in early-stage DKD (54). From a methodological standpoint, techniques such as liquid chromatography-mass spectrometry (LC-MS) facilitate the quantification of thousands of metabolites in urine samples (55). When coupled with machine learning algorithms (e.g., random forests), bioinformatics helps identify diagnostically informative combinations of characteristic metabolites (e.g., specific ratio alterations between tricarboxylic acid cycle intermediates and gut microbiota-derived metabolites) (46). Integrative analysis using both bioinformatics and AI not only aids in biomarker discovery but also contributes to the stratification of DKD into distinct molecular subtypes.

In the realm of AI, its impact is particularly evident in medical image analysis and predictive modeling. Deep learning-based systems, including convolutional neural networks, are now capable of automatically segmenting and quantitatively analyzing renal ultrasound images (56). These systems precisely measure kidney dimensions and volume while extracting textural features of the renal cortex through techniques like gray-level co-occurrence matrix analysis, translating subjective “echogenicity” into objective quantitative metrics. More importantly, AI enables the development of multimodal fusion models, algorithms such as gradient-boosting decision trees can be trained using diverse inputs, including imaging-derived features (e.g., cortical thickness), clinical parameters (e.g., urine protein-to-creatinine ratio), and omics-based biomarkers (57). Such models not only assist in diagnosis but also support personalized prognosis prediction. For instance, it can accurately estimate a patient's risk of experiencing a >50% decline in eGFR within 3 years.

In summary, bioinformatics extracts key molecular insights from large-scale datasets, while AI integrates these features with clinical and imaging data to build practical tools for precision diagnosis and prognostication. Synergistic use of bioinformatics and AI provides a concrete technical framework for early DKD detection and individualized intervention. Critically, the combined use of bioinformatics and AI is shifting DKD diagnosis from reliance on conventional markers toward a multi-omics driven precision prediction model that incorporates inflammatory signatures. Through the construction of multimodal fusion models, future applications may enable non-invasive, dynamic assessment of inflammatory activity and disease trajectory in patients. This prospect offers unprecedented support for the precise selection of anti-inflammatory treatment timing and targets, as well as for real-time monitoring of therapeutic response.

## 4 New advances in treatment

In recent years, SGLT2 inhibitors and ns-MRAs (e.g., Finerenone) have emerged as novel therapeutic strategies with significant renoprotective benefits. Their mechanisms extend beyond conventional glycemic or blood pressure control, with anti-inflammatory and antifibrotic properties representing core components of their efficacy. SGLT2 inhibitors alleviate glomerular hypertension and hyperfiltration by inhibiting the tubular reabsorption of glucose and sodium. They also improve mitochondrial function and reduce oxidative stress, suppressing inflammatory responses at the level of energy metabolism (58). On the other hand, Finerenone as a ns-MRA directly targets the overactivated mineralocorticoid receptor (MR) which is persistently stimulated by hyperglycemia and other risk factors in DKD. Activation of MRs leads to upregulation of pro-inflammatory and pro-fibrotic genes. By antagonizing MRs, Finerenone directly attenuates renal inflammation and fibrosis. It also confers multiple vascular and renal protective effects. For example, Finerenone is found to reduce endothelial cell apoptosis, inhibit smooth muscle cell proliferation, and limit inflammatory responses following vascular injury, facilitating endothelial repair and preventing maladaptive vascular remodeling (59).

In addition to theoretical feasibility, therapeutic value of both SGLT2 inhibitors and ns-MRAs is supported by robust clinical evidence. Both the CREDENCE trial (SGLT2 inhibitors) and the FIDELIO-DKD study (Finerenone) demonstrate that when added to standard renin-angiotensin system (RAS) blockade, these agents significantly reduce the risk of renal function decline and ESRD in patients with DKD (60, 61). Importantly, renoprotective effects of these agents are independent from glucose-lowering or blood pressure-lowering actions. Hence, SGLT2 inhibitors and Finerenone likely act directly on anti-inflammatory and antifibrotic pathways. The introduction of these medications marks a paradigm shift in DKD management, moving beyond risk factor control and toward direct targeting of pathophysiological processes.

Another important therapeutics, GLP-1RAs, has also been shown to provide renoprotection independent from glycemic control, partly due to their anti-inflammatory properties. Studies indicate that GLP-1RAs activate the cAMP/PKA signaling pathway and suppress key inflammatory mediators such as NF-κB in both renal and immune cells, thereby decreasing the production of pro-inflammatory cytokines (62). *Post hoc* analyses of major cardiovascular outcome trials (e.g., LEADER, REWIND) further revealed that GLP-1RAs significantly lower the risk of composite renal events in diabetic patients (63, 64). Thus, GLP-1RAs, together with SGLT2 inhibitors and Finerenone, form the cornerstone of a contemporary therapeutic framework aimed at mitigating DKD progression via anti-inflammatory mechanisms.

Beyond established agents described above, drug development is progressing along two major avenues. The first one involves targeted therapies informed by precision medicine, including specific NLRP3 inflammasome inhibitors (e.g., MCC950) and IL-1β antagonists (e.g., Canakinumab) (65–67). These agents are designed to precisely disrupt specific pathogenic inflammatory cascades, potentially offering enhanced efficacy and a more favorable safety profile. A key challenge in this area, however, is the achievement of kidney-selective drug delivery which enables effective local suppression of renal inflammation while minimizing systemic immune disruption. Overcoming this targeting barrier is essential for the successful clinical translation of similar therapies.

Simultaneously, natural products hold considerable promise in DKD drug development due to their multi-targeting, anti-inflammatory, and antioxidant properties (68–70). Extensive research has demonstrated that various natural bioactive compounds (e.g., flavonoids, alkaloids) exert synergistic protective effects by modulating multiple pathogenic pathways. Specifically for anti-inflammation, certain natural products directly inhibit NLRP3 inflammasome activation or antagonize the NF-κB signaling pathway, reducing the release of pro-inflammatory factors (71, 72). With respect to antifibrotic and cellular homeostasis regulation, natural products mitigate DKD progression through multidimensional mechanisms, including interference with the TGF-β/Smad pathway, activation of the Nrf2/ARE pathway, and restoration of podocyte autophagy (73–76). Critically, the integration of multi-omics technologies provides powerful tools for systematically deciphering complex actions of natural products, advancing relevant research from a “whole-system” model toward "precision targeting” and establishing a scientific foundation for the development of standardized therapies (77).

Of particular significance, the broad cardiorenal benefits observed with these treatments reinforce the concept of DKD as a manifestation of diabetes-associated panvascular disease. By intervening shared pathological pathways—such as inflammation and fibrosis—across the cardiovascular and renal systems, novel therapies enable synergistic management of multiple diabetic complications. This integrated approach represents a critical future direction not only for DKD treatment but also for comprehensive complication management in diabetes. Key anti-inflammatory and anti-fibrotic mechanisms of the therapeutics discussed above are summarized in Table 2.

**Table 2:** Key anti-inflammatory and anti-fibrotic mechanisms of pharmacologic interventions in diabetic kidney disease.

**Drug class**	**Experimental model**	**Key anti-inflammatory/anti-fibrotic mechanism**	**Dosage/concentration**	**References**
SGLT2 Inhibitors (e.g., Canagliflozin)	Patients with type 2 diabetes mellitus (T2DM) and CKD (CREDENCE trial)	Attenuates glomerular hypertension and improves mitochondrial function, thereby suppressing inflammation at the metabolic source;	100 mg, once daily	(58, 60)
ns-MRAs (e.g., Finerenone)	Patients with T2DM and CKD (FIDELIO-DKD trial)	Antagonizes the MR to inhibit pro-inflammatory and pro-fibrotic signaling, reducing endothelial apoptosis, inhibiting smooth muscle cell proliferation, attenuating leukocyte recruitment, and promoting vascular repair;	10 or 20 mg, once daily	(59, 61)
GLP-1 Receptor Agonists (e.g., Liraglutide, Dulaglutide)	Patients with T2DM (Cardiovascular outcome trials)	Activates the cAMP/PKA pathway, inhibiting key inflammatory signaling (e.g., NF-κB) and reducing pro-inflammatory cytokine production;	Liraglutide: 1.8 mg/day Dulaglutide: 1.5 mg/week	(62–64)
Targeted Therapies (Preclinical or Halted) (e.g., MCC950, Canakinumab)	Preclinical rodent models of DKD	Specifically inhibits NLRP3 inflammasome assembly and neutralizes IL-1β activity, thereby precisely blocking specific inflammatory pathways;	Preclinical concentrations	(65–67)
Natural Products (e.g., Flavonoids, Alkaloids)	Preclinical rodent models of DKD	Multi-targeted actions: modulates key signaling pathways including TGF-β/Smad, NF-κB, and NLRP3 to exert synergistic anti-inflammatory and anti-fibrotic effects;	Varies by specific compound	(68–76)

## 5 Discussion

Despite substantial advances on multiple fronts—from understanding inflammatory mechanisms to developing diagnostics and anti-inflammatory therapies—numerous challenges and opportunities remain for DKD research. Firstly, inflammatory pathways in DKD is a highly complex and interconnected network. Future studies should extend beyond the description of isolated pathways to elucidate specific crosstalks and hierarchical relationships between key signaling axes—such as those involving the NLRP3 inflammasome and NF-κB. It is particularly important to determine whether these interactions vary among different renal intrinsic cells and infiltrating immune cells, with clarifying respective underlying mechanisms. For example, identifying cell types in which NF-κB serves as an essential “priming” signal for NLRP3 activation, and how following released IL-1β feeds back to modulate NF-κB in different cellular contexts, would help delineate the inflammatory amplification loops in DKD. Clarifying such cell-type-specific interaction network is fundamental to understand the central inflammatory processes in DKD, and developing precise therapies.

Secondly, constructing a molecular inflammatory subtyping system for DKD based on existing inflammatory biomarkers and integrating it with multi-omics technologies and AI, will be immensely helpful for advancing precision medicine. Future therapeutic approaches may thus evolve beyond the “one-size-fits-all“ model toward tailored regimens—selecting optimal combinations of drugs (SGLT2 inhibitors, Finerenone, or other investigational anti-inflammatory agents) according to specific inflammatory subtype.

Moreover, exploring novel therapies that target innate and adaptive immune responses—such as cytokine-directed biologics and regulatory T cell based strategies—holds considerable promise. Natural product derived bioactive compounds also offer unique potential for modulating complex inflammatory networks due to their multi-target properties. Future research should actively investigate the response to treatment containing both natural products and conventional standard therapies. Synergistic mechanisms by which the combined therapy mitigates DKD should also be systematically elucidated using multi-omics approaches.

Critically, future investigations must transcend a kidney-centric view and adopt a broader systemic perspective. DKD represents the renal manifestation of diabetic panvascular disease. Its pathogenesis and progression are profoundly influenced by systemic inflammation and inter-organ crosstalk. Therefore, further exploration of the molecular mechanisms governing “organ–organ dialogue”—such as the gut-kidney, oral-kidney, and heart-kidney axes—will be essential to unraveling the systemic nature of DKD. For instance, elucidating how gut microbiota-derived metabolites or orally sourced inflammatory mediators remotely regulate the renal immune microenvironment may reveal novel therapeutic targets. Findings from this research would also provide a rationale for precision modulation of the gut-kidney axis via dietary interventions, probiotics, or natural product-derived drugs.

To this end, integrated therapeutic strategies capable of simultaneously targeting multiple organs and pathways should be developed. These strategies should not only act directly on the kidneys but also aim to reduce systemic inflammatory burden and preserve vascular integrity. Successful development of such treatment enables fundamental prevention and control of diabetic panvascular complications. Finally, translating these preclinical insights into clinical practice will require validation through large-scale prospective studies and real-world evidence to evaluate the long-term benefits and safety. Optimal timing to apply different anti-inflammatory strategies across disease stages should also be determined.

In summary, this review highlights the importance of understanding DKD within a systemic inflammation and panvascular disease framework by examining its inflammatory mechanisms, emerging diagnostic approches, and new advance in treatments. Looking forward, a core strategy centered on anti-inflammation, combining with precise molecular subtyping, multi-target integrated interventions, and a deepened understanding of inter-organ mechanisms represents a critical pathway to ultimately overcome DKD.
